# Partial Recovery of Respiratory Function and Diaphragm Reinnervation following Unilateral Vagus Nerve to Phrenic Nerve Anastomosis in Rabbits

**DOI:** 10.1371/journal.pone.0079552

**Published:** 2013-11-12

**Authors:** Junxiang Wen, Mingjie Yang, Lijun Li, Guixin Sun, Jun Tan

**Affiliations:** 1 Department of Orthopaedics, Shanghai East Hospital, Tongji University School of Medicine, Shanghai, China; 2 Department of Emergency, Shanghai East Hospital, Tongji University School of Medicine, Shanghai, China; Emory University, United States of America

## Abstract

Respiratory dysfunction is the leading cause of mortality following upper cervical spinal cord injury (SCI). Reinnervation of the paralyzed diaphragm via an anastomosis between phrenic nerve and a donor nerve is a potential strategy to mitigate ventilatory deficits. In this study, anastomosis of vagus nerve (VN) to phrenic nerve (PN) in rabbits was performed to assess the potential capacity of the VN to compensate for lost PN inputs. At first, we compared spontaneous discharge pattern, nerve thickness and number of motor fibers between these nerves. The PN exhibited a highly rhythmic discharge while the VN exhibited a variable frequency discharge pattern. The rabbit VN had fewer motor axons (105.3±12.1 vs. 268.1±15.4). Nerve conduction and respiratory function were measured 20 weeks after left PN transection with or without left VN-PN anastomosis. Compared to rabbits subjected to unilateral phrenicotomy without VN-PN anastomosis, diaphragm muscle action potential (AP) amplitude was improved by 292%, distal latency by 695%, peak inspiratory flow (PIF) by 22.6%, peak expiratory flow (PRF) by 36.4%, and tidal volume by 21.8% in the anastomosis group. However, PIF recovery was only 28.0%, PEF 28.2%, and tidal volume 31.2% of Control. Our results suggested that VN-PN anastomosis is a promising therapeutic strategy for partial restoration of diaphragm reinnervation, but further modification and improvements are necessary to realize the full potential of this technique.

## Introduction

Spinal cord injury (SCI) is a devastating condition resulting in permanent disability or mortality depending on the injury level. Approximately 40% of SCIs occur at the cervical level [1], resulting in respiratory function deficits. For those patients with upper cervical SCI, mechanical ventilation (MV) is still the routine choice to sustain life, which is associated with a number of serious side effects, including infection, atelectasis and diaphragm muscle atrophy [2]–[4]. Phrenic nerve pacing (PNP) has significantly improved the quality of life in patients with ventilator-dependent tetraplegia [5]–[8]. However, a substantial number of patients with mid-cervical (C3-5) spinal cord injury are not candidates for successful PNP [5], [9], [10]. Thus, a series of alternative techniques such as intercostal muscle pacing [9], [11]–[13], combined intercostal and phrenic nerve pacing [14], and intercostal to phrenic transfer with diaphragmatic pacing [15] have been introduced. For all these interventions, however, the pattern and level of ventilation induced by current pacing systems are fixed and not amenable to physiological control to match metabolic need [5], [16].

Reports demonstrated that diaphragm reinnervation through a donor nerve and phrenic nerve (PN) anastomosis is a potential strategy to restore respiratory autonomy and obviate external electronic pacing. Neurotization of the hypofunctional PN by the spinal accessory nerve (SAN) has been proposed as a possible way to restore physiological respiration [17]–[20]. In cats and rats with experimental PN transection and respiratory deficits, effective inspiratory recovery was observed following an anastomosis of the PN and recurrent laryngeal nerve (RLN) anastomosis [21]–[23]. However, in rabbit model, the same treatment resulted in poor inspiratory activity during quiet breathing [24].

Studies have suggested that the vagus nerve (VN) contains fibers that produce high amplitude efferent discharges synchronous with PN during inspiration and expiration [25], [26], and the frequency of these discharges increase or decrease in parallel with discharge of the PN during gasping [27]. In rats, Guth et al. [26] demonstrated regeneration of vagal fibers into the PN, keeping a large number of end-plates in the hemidiaphragm on the surgical side that were morphologically indistinguishable from those on the contralateral side, and inspiratory volleys were recorded from the anastomosed PN. In addition, contraction of the initially paralyzed hemidiaphragm was observed after VN-PN anastomosis in rats and dogs [26], [28]. However, in these animal studies, respiratory function has not been assessed after nerve anastomosis, and the effectiveness of respiratory recovery is still unknown.

In the present study, we first compared spontaneous discharges, nerve thickness, and number of motor fibers between the rabbit VN and PN to assess the suitability of the VN to replace transected PN to innervate the diaphragm. We then investigated the effectiveness of VN-PN anastomosis for respiratory recovery in rabbits with unilateral phrenicotomy by testing respiratory function and compound muscle action potentials (CMAPs) after surgery.

## Methods

### 1. Ethical approval and animals

New Zealand rabbits were obtained from the Animal Center of Fudan University, Shanghai, China. They were maintained at 22°C on a 12 h light/dark cycle with free access to rabbit chow and water. All animal experiments were conducted in accordance with protocols approved by the Institutional Animal Care Committee of Fudan University.

### 2. Animals and groups

**Figure 1:**
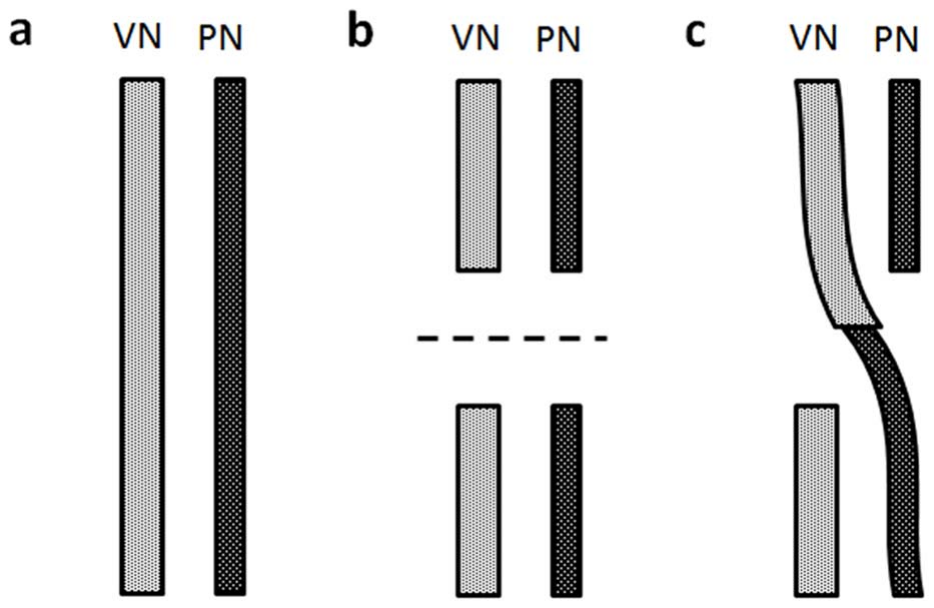
Schematic showing the surgical preparations in the three groups. **A.** Control Group: Both VN and PN were intact. **B.** Transection Group: Both VN and PN were transected. **C.** Bridge group: The proximal end of VN was anastomosed to the distal end of the PN.

A total of 22 male New Zealand rabbits (4 months old) were included in this study. To exclude sex differences in respiratory flow and tidal volume, only male animals were used. Four animals were used for comparison of spontaneous efferent discharge, nerve thickness, and motor fiber number between the vagus nerve (VN) and phrenic nerve (PN). The remaining animals were divided into three equal groups (Figure 1): a Control Group in which both the left VN and PN were exposed but not transected, a Transection Group in which both left VN and PN were transected 1.5 cm rostral to the clavicle, and a Bridge Group in which both left VN and PN were transected 1.5 cm rostral to the clavicle, and the rostral cut end of the VN was immediately anastomosed to the caudal cut end of the PN.

### 3. Anesthesia and surgical procedure

Animals were anesthetized using 2% pentobarbital (1.5 ml/kg) injected through the marginal ear vein. The surgical procedure was performed with the animals in a supine position via a ventral medial approach. The VN was exposed in the carotid sheath running parallel to the lateral side of the carotid artery. The PN was exposed on the surface of the anterior scalene muscle, and was identified by abdominal expansion induced by electrical stimulation (0.5 mA) (SMUP-U System, Fudan University, Shanghai, China). In brief, the putative PN was stimulated by single 0.5 mA pulses and identity confirmed by evoked abdominal expansion resulting from diaphragm contraction. In Control Group, both VN and PN were exposed but left intact. In the Transection Group, left VN and PN were transected and a 5 mm segment of both nerves removed at a level 1.5 cm higher than the clavicle to prevent regeneration. In the Bridge Group, VN and PN were transected 1.5 cm higher than the clavicle and the rostral cut end of the VN anastomosed to the caudal cut end of the PN by epineurial suturing with 10.0 nylon thread (Alcon) under a surgical microscope (SXP-1B, Medical Optical Company, Shanghai, China). After cleaning with iodine and saline, the incision was closed without drainage. All animals received post-operative analgesic treatment for three days (Buprenorphine, 0.02 mg/kg, subcutaneous injection, Yangzhou No.3 Pharmaceutical Co., Ltd, China).

### 4. Spontaneous efferent discharges from VN and PN

The four rabbits reserved for comparison of VN versus PN spontaneous discharges, morphology, and motor axon content, were anesthetized using 20% urethane (4 ml/kg). Both VN and PN were carefully exposed under a surgical microscope (SXP-1B, Medical Optical Company, Shanghai, China). Nerve discharges were recorded using the SMUP-U system (Fudan University, Shanghai, China). Briefly, VN and PN were transected 1 cm rostral to clavicle, and the proximal end placed on a bipolar metal recording electrode respectively. Spontaneous discharges were band-pass filtered (160 Hz to 10 kHz), amplified (8000×), recorded, and analyzed using MFlab200 software (Fudan University, Shanghai, China).

### 5. Anatomic measurement of VN and PN

After measurement of spontaneous discharges in these 4 rabbits, distances between VN and PN on both sides were measured by digital caliper with precision of 0.01 mm (SF2000, Guilin Guanglu Measuring Instrument Co., Ltd, Guangxi, China) at a level 1.5 cm rostral to the clavicle. Then animals were sacrificed by anesthesia (20% urethane) overdose through the marginal ear vein. Nerve segments of VN and PN (3 mm) 1.5 cm higher than the clavicle were removed for diameters measurement with a portable reading microscope with a precision of 0.0025 mm (MJ-300XS, Meijing Electronic Instrument Co., Ltd, Shanghai, China).

### 6. Acetylcholinesterase (AChE) staining

After anatomic measurements, harvested nerve segments were fixed in 10% formalin for 48–72 h at 4 °C, transferred to 30% sucrose for 24–48 h at 4 °C, and then embedded in O.C.T (Fisher, PA, USA). Frozen transverse sections of 15 μm were cut on a freezing microtome (Bright OTF5000, SCILOGEX, USA), and every 5th section collected and mounted on slides. Slides were heated to 37 °C for 30 min then stored at –80 °C until AChE staining. AChE staining was performed according to Karnovsky and Roots [29]. Briefly, sections were immersed in an incubation medium consisting of iodized acetylthiocholine, potassium ferricyanide, and copper sulfate. Iodized acetylthiocholine acts as substrate for AChE, and the latter two chemicals react with the liberated thiocholine, producing a brownish precipitate. After incubation for 6 h at room temperature, the slides were lightly counterstained with Harris hematoxylin. Sections were examined using an imaging microscope (Carl Zeiss, Germany) and analyzed with ImageJ (NIH) by experimenters (M.Y. and L.L.) blind to the source nerve to determine the numbers of motor fibers.

### 7. Test of respiratory function

Twenty weeks after surgery, 18 animals in three groups (6/group) were anesthetized using 20% urethane (4 ml/kg) and the tracheal cartilage exposed from the cricoid cartilage to the fifth tracheal ring by a medial cervical approach. A reversed “T” shape slit was made in the anterior tracheal wall between the second and fourth tracheal ring and a “Y” shape endotracheal tube placed into the trachea. Respiratory air flow and tidal volume were measured using a pneumotachograph transducer (SKY Biological Signal Management System, Fudan University, Shanghai, China) directly connected to one end of the “Y” shape endotracheal tube. The records of respiratory air flow and tidal volume were then filtered (1.6 Hz to50 kHz), amplified (1000×), saved and analyzed using MFlab200 software (Fudan University, Shanghai, China).

### 8. Compound muscle action potential (CMAP) measurement

After measurements of respiratory function, VN and PN were exposed in the cervical area, and the diaphragm was exposed by laparotomy. Distal latencies (dLAT) and amplitudes of CMAP were measured using the Dantec Keypoint Evoked Potentials System (Dantec, Denmark). Briefly, the PN of Control Group rabbits, the proximal stump of the transected VN of Transection Group rabbits, or the VN rostral to the anastomosed site of Bridge Group rabbits were placed on a unipolar needle-stimulating electrode set to discharge single pulses of 0.5 mV for 0.1 ms at 1 Hz. A recording electrode was inserted into the center of the left hemi-diaphragm and a reference electrode inserted into the subcutaneous tissue between the stimulator and recording electrode. Five separate recording were acquired from each animal, saved, and analyzed as described.

### 9. Statistical Analysis

All results are expressed as mean ± standard deviation. Phrenic and vagus nerve parameters were compared by one-way analysis of variance (ANOVA). Multiple comparisons among the three groups were evaluated using Scheffe’s test (post-hoc analysis). A P < 0.05 was considered statistically significant. All data were analyzed statistically using SPSS 14.0 software (SPSS, Chicago, USA).

## Results

### 1. Spontaneous discharges of PN and VN

**Figure 2:**
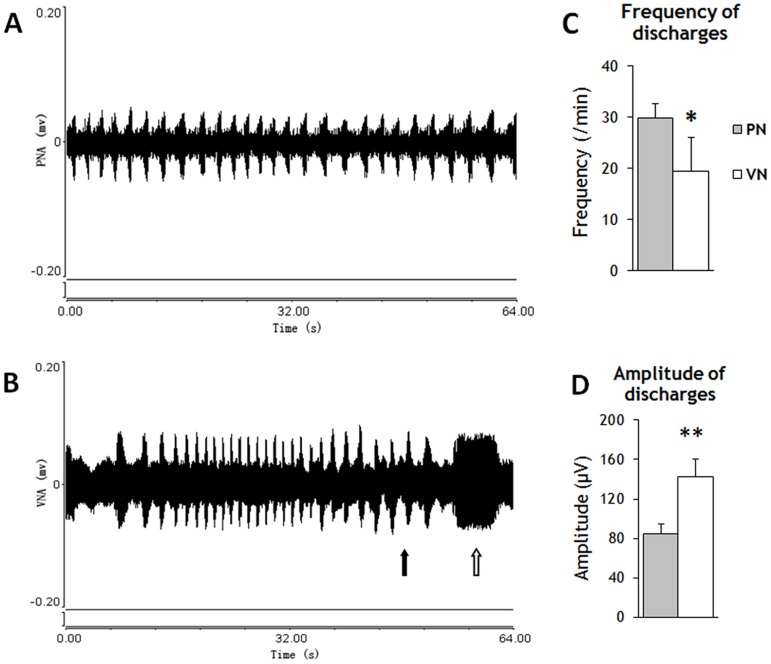
Spontaneous efferent discharges from the PN and VN. **A.** Rhythmic discharges from the PN. **B.** Sporadic, variable frequency discharges from the VN. The frequency was sometimes close to that of the PN (solid arrow). Prolonged discharges (open arrow) were only recorded from the VN. **C.** Mean discharge frequencies from the PN and VN (p = 0.021). **D.** Discharge amplitudes of PN and VN (P<0.001). The error bars indicate standard deviation (SD). * p<0.05; ** p<0.001

Spontaneous discharges of the PN and VN were recorded and compared (Figure 2). The discharge frequency of the PN and VN was 29.2±2.9/min and 19.4±6.7/min, respectively. Frequency of the PN was highly rhythmic (Figure 2A), while VN varied markedly over time (Figure 2B). In addition, individual volleys recorded from the PN were of relatively uniform duration, whereas the VN exhibited occasional prolonged individual volleys (open arrow in Figure 2B). There was a significant difference in mean discharge frequency between PN and VN (p = 0.021). On occasion, however, the frequency of VN discharge approached to that of the PN (solid arrow in Figure 2B). The mean amplitude of discharges of VN was higher than that of PN (142.1±18.6 µV vs. 84.4±10.7 µV; P<0.001).

### 2. Diameters of PN and VN, and distances between them

There was no significant difference in the diameters of left and right PN or VN (p>0.05), so bilateral diameters were compared together (Table 1). The VN was 1.67 times thicker than the PN (1.2934±0.1085 mm vs. 0.7729±0.0716 mm; p = 0.003). The distances between PN and VN of the left and right sides were 6.754±1.113 mm and 7.343±1.052 mm, respectively (P>0.05).

**Table 1:** Diameters of the PN and VN and the distances between them (–x ± SD,mm)

	Left	Right	Average
PN	0.7836±0.0738	0.7621±0.0694	0.7729±0.0716
VN	1.2856±0.0958	1.3012±0.1211	1.2934±0.1085
Distances between PN and VN	6.754±1.113	7.343±1.052	―

There was no significant difference in nerve diameter between left and right sides for either nerve (P>0.05), so we calculated the average diameter using data from both left and right nerves. The VN was 1.67 times thicker than the PN (P = 0.003).

### 3. Numbers of motor fiber of PN and VN

**Figure 3:**
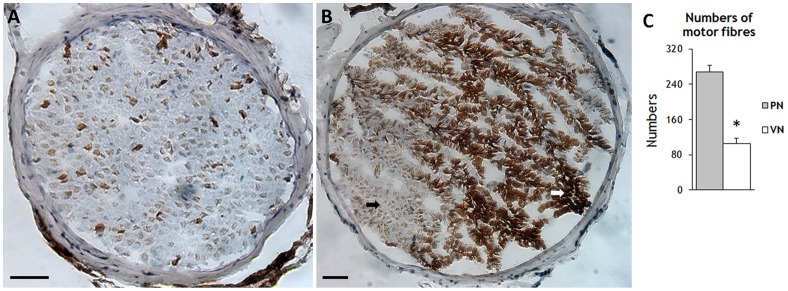
AChE staining of cross-section of the PN and VN (×200). **A,** AChE staining of the PN. Almost all fibers presented the typical characteristics of motor fibers, with AChE-stained axons and unstained myelin sheaths. **B**, AChE staining of the VN. Only some areas presented the typical characteristics of motor fibers (black arrow), while other fibers were intensely and cloddy stained (white arrow), indicative of preganglionic fibers. Scale bar represent 25 μm in A and B. **C**, Comparison of motor fiber numbers in the PN and VN. The error bars indicate standard deviation (SD). * p<0.001

Acetylcholinesterase (AChE) staining of PN and VN cross-sections were performed and the number of motor fibers was counted (Figure 3). Almost all fibers of the PN showed the typical morphological and histochemical properties of motor fibers (Figure 3A), with axons stained brown for AChE enveloped by unstained myelin sheaths. However, only subregion of the VN showed typical property of motor fibers positive for AChE staining (Fig 3B), and majority of VN fibers showed intense cloddy staining, which are unmyelinated (or lightly myelinated) preganglionic fibers. Twenty images from each nerve specimen were analyzed using ImageJ by researchers (M.Y. and L.L.) blind to nerve identity to estimate the number of motor fibers. There were no significant differences between left and right side for either the PN (269.3±17.2 vs. 266.8±14.7; P>0.05) or VN (108±13.8 vs. 102.7±10.7; P>0.05). However, the bilateral mean number of PN motor fibers was significantly higher relative to VN motor fibers (268.1±15.4 vs. 105.3±12.1; P<0.001).

### 4. Respiratory air flows and tidal volume

**Figure 4:**
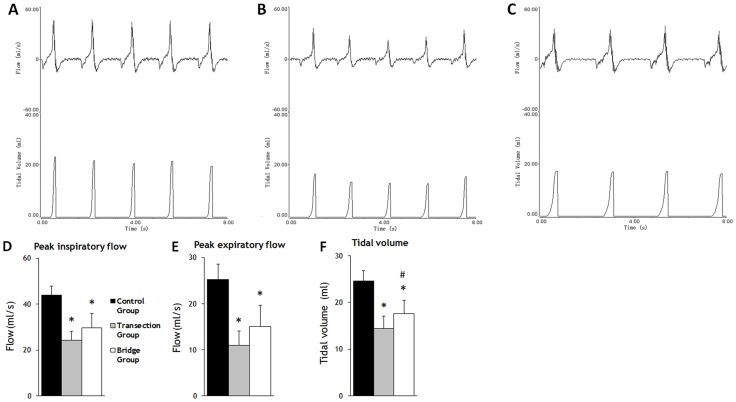
Records of respiratory air flow and tidal volume from Control Group (A), Transection Group (B) and Bridge Group (C) rabbits 20 weeks after surgery. Flow curves above the base line indicate the inspiratory phase, below the expiratory phase. **D**, Comparison of peak inspiratory flow (PIF). **E**, Comparison of peak expiratory flow (PEF). **F**, Comparison of tidal volume. The error bars indicate standard deviation (SD). * p<0.001 vs. Control Group; # p<0.05 vs. Transection Group

20 weeks after surgery, compared to Control Group, respiratory air flow and tidal volume revealed marked reductions in the Transection Group and partial recovery in the Bridge Group (Figure 4).

The mean peak inspiratory flow (PIF) was 43.82±3.92 ml/s in the Control Group, 24.26±3.9 ml/s in the Transection Group, and 29.74±6.08 ml/s in the Bridge Group (Figure 4D). The Control Group PIF was 1.8 times higher than the Transection Group PIF (P<0.001), while the Bridge Group PIF was 0.68 times that of the Control Group (P<0.001) and 1.23 times that of the Transection Group (P = 0.051). The Bridge Group PIF was improved by 22.6% compared to Transection Group. Thus, there was a trend for partial recovery, but improved PIF was only 28.0% of control ((Bridge – Transection)/(Control - Transection) × 100%) 20 weeks after VN-PN anastomosis.

The mean peak expiratory flow (PEF) was 25.25±3.32 ml/s in the Control Group, 11.01±3.06 ml/s in the Transection Group, and 15.02±4.65 ml/s in the Bridge Group (Figure 4E). The Control Group PEF was 2.3 times higher than the Transection Group PEF (P<0.001), while the Bridge Group PEF was 0.59 times that of the Control Group (P<0.001) and 1.36 times that of the Transection Group (P = 0.074). The Bridge Group PEF was improved by 36.4% compared to Transection Group. Thus, again there was a trend for partial recovery, but improved PEF was only 28.2% of control ((Bridge – Transection)/(Control - Transection) × 100%).

The mean tidal volume was 24.58±2.29 ml in the Control Group, 14.47±2.66 ml in the Transection Group, and 17.62±2.89 ml in the Bridge Group (Figure 4F). The Control Group tidal volume was 1.7 time that of the Transection Group (P<0.001), while the Bridge Group tidal volume was 0.72 times that of the Control Group (P<0.001) and 1.22 times that of the Transection Group (p = 0.041). The Bridge Group was improved by 21.8% compared to Transection Group. Thus, there was a partial improvement in tidal volume following VN-PN anastomosis, suggesting that some VN fibers did successfully innervate the diaphragm, but improved tidal volume was only 31.2% of control ((Bridge – Transection)/(Control - Transection) × 100%).

### 5. Distal latencies (dLAT) and amplitude of compound muscle action potential (CMAP)

**Figure 5:**
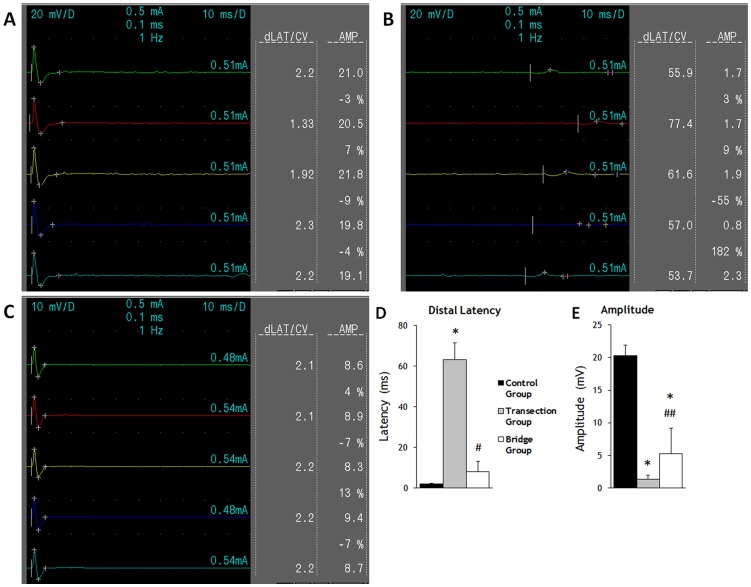
Records of CMAP from Control Group (A), Transection Group (B) and Bridge Group (C) rabbit showing both distal latency (dLAT) and amplitude 20 weeks after surgery. The stimulator electrode was place on the intact VN in Control Group, the proximal end of the transected VN in the Transection Group, and the VN rostral to the anastomosed site in the Bridge Group. **D**, Comparison of dLAT. **E**, Comparison of amplitude. The error bars indicate standard deviation (SD). * p<0.001 vs. control group; # p<0.001 vs. Transected Group; ## p<0.05 vs. Transected Group

To ascertain the extent of diaphragm innervation by the VN, we measured dLAT and CMAP amplitude (Figure 5). The dLAT, as measured from the time of nerve stimulation to the first deflection from baseline of the muscle action potential (AP) response, was 2.016±0.410 ms in the Control Group, 63.025±8.305 ms in the Transection Group, and 7.925±5.134 ms in the Bridge Group. The Transection Group dLAT was 30 times that of the Control Group (P<0.001), while the Bridge Group was 4 times that of the Control Group (P = 0.136) and 0.13 times that of the Transection Group (P<0.001). The Bridge Group was improved by 695% compared to Transection Group. Thus, anastomosis significantly recovered the neural transmission time to the left hemidiaphragm.

The diaphragm AP amplitude was 20.3±1.587 mV in the Control Group, 1.338±0.650 mV in the Transection Group, and 5.250±3.966 mV in the Bridge Group. The Control Group amplitude was 15 times that of the Transection Group (P<0.001), while the Bridge Group amplitude was 0.25 times that of the Control Group (P<0.001) and 4 times that of the Transection Group (P = 0.018). The Bridge Group was improved by 292% compared to Transection Group. This significant increase in diaphragm AP amplitude following anastomosis suggests that the VN did indeed partially innervate the denervated left hemidiaphragm.

## Discussion

Significant differences in spontaneous discharge, nerve thickness, and motor fiber number were found between the vagus nerve (VN) and phrenic nerve (PN). Despite this, we found that VN fibers could regenerate into the PN after VN-PN anastomosis and partially ameliorate respiratory dysfunction in rabbits with unilateral phrenicotomy. However, recovery was not sufficient to sustain respiration after contralateral phrenicotomy. Even though, as we know, a little recovery of function is still of significance when it comes to compromised function in individuals with spinal cord injury (SCI).

### 1. Comparisons between VN and PN

Spontaneous efferent discharges of the VN and PN differed in both frequency and amplitude. The frequency of PN discharge was constant, while VN discharges varied in both frequency and duration of individual volleys. The PN is responsible for respiration, which is of constant frequency when metabolism is stable. In contrast, the VN provides mixed innervation to the heart, lung, and a greater part of the digestive tract and other abdominal viscera [30], so VN discharge frequency varies according to the needs of these different systems. The long duration volleys observed in the present study may be outputs to the digestive tract. Nonetheless, VN discharge frequency was occasionally similar to that of the PN, an observation in accord with other studies reporting efferent discharges from the VN synchronous with respiration [25]–[27]. It has been suggested that VN volleys in phase with inspiration are from recurrent laryngeal fibers [26]. However, reinnervation of the cat diaphragm with spinal accessory, facial, or long thoracic nerves did not lead to functional recovery [20]. This is not surprising given that there is no evidence that any of these nerves carry respiratory volleys [26]. We found that the mean amplitude of a VN volley was 1.7 times larger than that from the PN. It is possible then that partial respiratory recovery seen in the VN-PN anastomosis is due to the generation of spontaneous efferent discharges.

In the cervical area, the mean distance between the VN and PN was less than 8 mm, so anastomosis can be performed without excessive tension (even though the rabbit VN is 1.67 times thicker than the PN). The number of motor fibers in the rabbit cervical PN was about 2.5 times more than that in the VN. A study on human cadavers revealed that there were 3–4 times more motor fibers in the PN compared to VN [31]. Research in rats suggested that it may be possible to obtain a high number of regenerative myelinated axons by connecting a proximal donor nerve with fewer axons to a distal nerve stump [32]. However, 80%–90% of the axons in the VN are afferent [33]. Thus, much of the contact area between VN and PN after anastomosis would be taken up by VN sensory fibers, and the available area for motor fibers to grow into the PN would be severely limited. We found that motor fibers were located mainly on one side of the VN, so it may be more efficient to use only the motor fiber side for anastomosis, although this may prove technically challenging.

### 2. Diaphragm reinnervation after VN-PN anastomosis

Animals in the Bridge Group showed an improvement in respiratory function compared to animals in Transection Group suggesting specific reinnervation by VN-PN anastomosis rather than other reinnervation [34]. Indeed, this was confirmed by enhanced muscle action potential in the diaphragm.

Unilateral cervical vagotomy without phrenicotomy could result in immediate and severe depression of respiratory frequency, with prolongation of the respiratory period and greatly enhanced inspiratory amplitude because of the abolished Hering-Breuer inflation reflex [35], [36]. However, a long-term study of dogs after bilateral vagotomy found that the prolonged inspiratory period lasted only 2 weeks [37]. In rats, however, transection of the VN resulted in only a mild increase in respiratory amplitude [26]. Studies in patients undergoing esophageal-carcinoma resection with or without sectioning of the right pulmonary vagal branch by lymphadenectomy found that both patient groups only had slight augmentation of breathing frequency and minute ventilation at rest two months after surgery [38]. Thus, it appears that the effect of vagotomy on respiration resolves gradually with time. In the present study, both PN and VN on left side were transected in the Transection Group, so the improvement of respiratory function in the Bridge Group was not due to the effect of vagotomy, but rather from VN-PN anastomosis.

Nerve conduction study (NCS) revealed improved nerve conduction through the VN-PN anastomosis as the distal latency (dLAT) in the Bridge Group was 0.13 times (P<0.001) that of the Transection Group, and 4 times (p = 0.136) that of the Control Group. Since an increased latency usually indicates damage to the myelin sheaths covering axons [39], [40], latency recovery to nearly normal in the Bridge Group indicates that at least some axons from the VN regenerated into the PN and were remyelinated. The compound muscle action potential (CMAP) amplitude in the Bridge Group was 0.25 times that of the Control Group (P<0.001) and 4 times that of the Transection Group (P = 0.018). Since CMAP amplitude reflects the functional status of myelinated axons with motor endplates [41], our result indicates that axons of the VN regenerated into the PN and reinnervated the hemidiaphragm. This is consistent with a histological study demonstrating that VN fibers regenerated into the PN after VN-PN anastomosis [26].

### 3. Functional efficiency of the reinnervated hemidiaphragm

In rabbits with inferior laryngeal nerve (ILN)-PN anastomosis, Derry et al. [24] found poor and neurogenic electrical inspiratory activity of the diaphragm during quiet breathing. However, electrical activity increased under acute hypoxia [24], [42]. Thus, poor respiratory functional recovery in the present study may be related to the poor electrical activity of the diaphragm under eupnea. An electrophysiological study in rats indicated that recurrent laryngeal nerve (RLN)-PN anastomosis resulted in functional diaphragm reinnervation similar to that of the intact contralateral side [23]. Although no measurements of respiratory function were shown, these authors demonstrated that rats with RLN-PN anastomosis could live for at least 7 days after section of the contralateral phrenic nerve or after complete section of the upper cervical spinal cord. However, a similar study in rabbit did not replicate these results [24], and the present study found that VN-PN anastomosis cannot fully restore respiration to the extent required for basic metabolism.

In the present study, we found that only a fraction of VN fibers are motor fibers and located to one side of the VN. Study suggested that the regeneration of VN fibers into the PN is likely nonselective [26]. Limited regeneration of motor fibers may be responsible for the only partial recovery observed after surgery. Presumably, selective anastomosis of the VN motor fibers alone could improve these results. Another possible method to improve respiratory outcome might be applying diaphragm pacing together with VN-PN anastomosis.

### 4. Disadvantages of VN-PN anastomosis

Although our results demonstrated that VN-PN anastomosis was able to improve respiratory function in rabbits with unilateral phrenicotomy, there are several disadvantages to this method. First, VN is a mixed nerve with branches to neck, chest, and abdomen, where it contributes to the innervation of the viscera and conveys sensory information of the organs to the central nervous system. So vagotomy in the cervical area would severe disrupt visceral function and homeostasis. Second, transection of the VN at the cervical level would lead to recurrent laryngeal nerve injury, which would result in hoarseness and even dyspnea because of the absence of movement on the affected side of the vocal cords [43], [44]. However, these symptoms might be significantly improved by laryngeal reinnervation [45], [46]. Third, 80%−90% of VN fibers are afferent (sensory) [30], so only 10%−20% of the surface area of anastomosis would be motor fibers that could regenerate into the PN and reinnervate the diaphragm. This is inefficient and cannot justify sacrifice of the other 80%−90% of the fibers in the VN. Hence, future studies should attempt anastomosis with only the VN motor fibers. End-to-side neurorrhaphy [47], [48] might also be a feasible method to save afferent fibers in the VN. However, it cannot guarantee that motor fibers in VN are precisely anastomosed into the PN because it is difficult to determine axonal organization during surgery. Thus, other more practical methods need to be developed.
